# *Anaplasma phagocytophilum* MSP4 and HSP70 Proteins Are Involved in Interactions with Host Cells during Pathogen Infection

**DOI:** 10.3389/fcimb.2017.00307

**Published:** 2017-07-05

**Authors:** Marinela Contreras, Pilar Alberdi, Lourdes Mateos-Hernández, Isabel G. Fernández de Mera, Ana L. García-Pérez, Marie Vancová, Margarita Villar, Nieves Ayllón, Alejandro Cabezas-Cruz, James J. Valdés, Snorre Stuen, Christian Gortazar, José de la Fuente

**Affiliations:** ^1^SaBio, Instituto de Investigación en Recursos Cinegéticos, Consejo Superior de Investigaciones Científicas, CSIC-UCLM-JCCMCiudad Real, Spain; ^2^Departamento de Sanidad Animal, Instituto Vasco de Investigación y Desarrollo Agrario (NEIKER)Derio, Spain; ^3^Biology Centre, Czech Academy of Sciences, Institute of ParasitologyČeské Budějovice, Czechia; ^4^Faculty of Science, University of South BohemiaČeské Budějovice, Czechia; ^5^UMR BIPAR, Animal Health Laboratory, INRA, ANSES, ENVAMaisons Alfort, France; ^6^Department of Virology, Veterinary Research InstituteBrno, Czechia; ^7^Department of Production Animal Clinical Sciences, Norwegian University of Life SciencesSandnes, Norway; ^8^Department of Veterinary Pathobiology, Center for Veterinary Health Sciences, Oklahoma State UniversityStillwater, OK, United States

**Keywords:** anaplasmosis, immunology, HL60, tick, vaccine, sheep, *Anaplasma phagocytophilum*

## Abstract

*Anaplasma phagocytophilum* transmembrane and surface proteins play a role during infection and multiplication in host neutrophils and tick vector cells. Recently, *A. phagocytophilum* Major surface protein 4 (MSP4) and Heat shock protein 70 (HSP70) were shown to be localized on the bacterial membrane, with a possible role during pathogen infection in ticks. In this study, we hypothesized that *A. phagocytophilum* MSP4 and HSP70 have similar functions in tick-pathogen and host-pathogen interactions. To address this hypothesis, herein we characterized the role of these bacterial proteins in interaction and infection of vertebrate host cells. The results showed that *A. phagocytophilum* MSP4 and HSP70 are involved in host-pathogen interactions, with a role for HSP70 during pathogen infection. The analysis of the potential protective capacity of MSP4 and MSP4-HSP70 antigens in immunized sheep showed that MSP4-HSP70 was only partially protective against pathogen infection. This limited protection may be associated with several factors, including the recognition of non-protective epitopes by IgG in immunized lambs. Nevertheless, these antigens may be combined with other candidate protective antigens for the development of vaccines for the control of human and animal granulocytic anaplasmosis. Focusing on the characterization of host protective immune mechanisms and protein-protein interactions at the host-pathogen interface may lead to the discovery and design of new effective protective antigens.

## Introduction

*Anaplasma phagocytophilum* (Rickettsiales: Anaplasmataceae) is an emerging tick-borne intracellular bacterial pathogen in many regions of the world, but vaccines are not available for prevention of transmission and infection in humans and animals (Dumler et al., 2001; Severo et al., 2013; Stuen et al., 2013, 2015; Bakken and Dumler, 2015). *Anaplasma phagocytophilum* causes human granulocytic anaplasmosis (HGA), which has emerged as a tick-borne disease of humans in the United States, Europe and Asia (Severo et al., 2013). In Europe, *A. phagocytophilum* is an established pathogen of small ruminants, most notably in sheep, where it was first described as the etiologic agent of tick-borne fever (TBF; Gordon et al., 1932; Foggie, 1951; Dugat et al., 2015). Clinical presentation of *A. phagocytophilum* infection has been also documented in goats, cattle, horses, dogs, cats, roe deer, and reindeer (Severo et al., 2013). Although, *A. phagocytophilum* is recognized as a threat for human and animal health in Europe and the United States, its pathogenic and epidemic potential is neglected in tropical regions of the world (Heyman et al., 2010; Dugat et al., 2015). Prophylactic uses of tetracycline together with acaricide applications for tick control are the main measures to control *A. phagocytophilum* infection in endemic areas (Woldehiwet, 2006; Stuen et al., 2015). However, these control measures raise concerns about their impact on the environment and human health, and the selection of resistant pathogens and ticks (Woldehiwet, 2006; Stuen et al., 2015).

Results using next generation sequencing technologies have advanced our understanding of the mechanisms by which *A. phagocytophilum* infection affects gene expression, protein content and microbiota in the vertebrate host and tick vector (Ge and Rikihisa, 2006; Sukumaran et al., 2006; de la Fuente et al., 2010, 2016a,b,c,d, 2017, Neelakanta et al., 2010; Rikihisa, 2011; Severo et al., 2012; Ayllón et al., 2013, 2015; Hajdušek et al., 2013; Villar et al., 2015a; Cabezas-Cruz et al., 2016, 2017; Gulia-Nuss et al., 2016; Abraham et al., 2017; Mansfield et al., 2017). However, less information is available on the bacterial molecules involved in pathogen infection and multiplication (Ge and Rikihisa, 2007; Huang et al., 2010; Lin et al., 2011; Troese et al., 2011; Mastronunzio et al., 2012; Oliva Chávez et al., 2015; Seidman et al., 2015; Villar et al., 2015b; Truchan et al., 2016). Definition of bacterial proteins involved in host-pathogen and vector-pathogen interactions may provide target antigens for the development of vaccines and therapeutics that interfere with pathogen host infection and transmission by ticks (Gomes-Solecki, 2014; de la Fuente and Contreras, 2015).

Recently, Villar et al. (2015b) demonstrated that *A. phagocytophilum* activates a new mechanism associated with bacterial cell stress and membrane proteins to counteract tick cell response to infection and favor pathogen infection and multiplication. Their results showed that *A. phagocytophilum* proteins, Major surface protein 4 (MSP4) and Heat shock protein 70 (HSP70), are localized on the bacterial membrane where they interact with a possible role during pathogen infection in ticks (Villar et al., 2015b). Furthermore, antibodies against MSP4 and HSP70 inhibited pathogen infection of tick cells, supporting that these proteins are involved in tick-pathogen interactions (Villar et al., 2015b). They proposed that the inhibitory effect of anti-MSP4 and anti-HSP70 antibodies could be the result of the antibodies blocking the interaction between bacterial ligands (e.g., MSP4) and tick receptors or an effect on proteins functionally important for bacterial infection and/or multiplication in tick cells (e.g., HSP70 and those physically and/or functionally interacting with it; Villar et al., 2015b). The results of these experiments suggested that *A. phagocytophilum* MSP4 and HSP70 proteins constitute candidate protective antigens to interfere with pathogen infection in the tick vector, *Ixodes scapularis*.

The characterization of the *A. phagocytophilum* proteome demonstrated that chaperones, surface and stress response proteins are among the most abundant proteins found in *I. scapularis* tick salivary glands (Mastronunzio et al., 2012). HSP70 is a chaperone involved in protein folding and stress response (Johnson, 2012). This protein functions by protecting cells from stress-induced lethal damage and under physiological growth conditions by acting as carriers for immunogenic peptides, assisting in protein export or mediating adherence to host cells and may play an essential role during cell division (Scopio et al., 1994; Susin et al., 2006; Multhoff, 2007; Seydlová et al., 2012). The role of MSPs such as MSP4 in adhesion to tick cells for bacterial infection has been demonstrated in *A. marginale* (de la Fuente et al., 2001) and *A. phagocytophilum* (Villar et al., 2015b).

*Anaplasma phagocytophilum* infects vertebrate host neutrophils and various tick tissues, where it develops within membrane-bound inclusions in the cell cytoplasm (Severo et al., 2013). However, this pathogen has evolved common molecular mechanisms to establish infection in tick vectors and vertebrate hosts that collectively mediate pathogen infection, development, persistence, and survival (de la Fuente et al., 2016a). These strategies include, but are not limited to, remodeling of the cytoskeleton, inhibition of cell apoptosis, manipulation of the immune response, and the use of rickettsial proteins for infection and manipulation of tick and host gene expression (Cabezas-Cruz et al., 2016; de la Fuente et al., 2016a).

Based on these results, we hypothesized that the use of common strategies by *A. phagocytophilum* to establish infection in ticks and vertebrate hosts resulted in similar functions for MSP4 and HSP70 proteins in host-pathogen and tick-pathogen interactions. To address this hypothesis, in this study we characterized the role of these bacterial proteins in infection of vertebrate host cells and their potential protective capacity in immunized sheep. The results showed that *A. phagocytophilum* MSP4 and HSP70 are involved in host-pathogen interactions during pathogen infection, but were only partially protective against pathogen infection in sheep.

## Materials and methods

### Ethics statement

The study was ethically approved by the local Animal Health and Welfare Authority (Diputación Foral de Alava) with reference No. 1820, 12th May 2015, following Spanish ethical guidelines and animal welfare regulations (Real Decreto 53/2013).

### Production of recombinant proteins and rabbit antibodies

The His-tag recombinant *A. phagocytophilum* human NY18 isolate (Asanovich et al., 1997) proteins MSP4 (AFD54597) and HSP70 (KX891324) were produced in *Escherichia coli* BL21 cells (Champion pET101 Directional TOPO Expression kit, Carlsbad, CA, USA), after induction with IPTG and purified using the Ni-NTA affinity column chromatography system (Qiagen Inc., Valencia, CA, USA) as previously described (Villar et al., 2015b). Recombinant purified proteins showed purity higher than 85% of total proteins and were used to immunize rabbits to purify IgGs from preimmune and immunized animals (Montage Antibody Purification Kit and Spin Columns with PROSEP-A Media, Millipore, Billerica, MA, USA) for Western blot and antibody inhibition analyses as previously described (Villar et al., 2015b).

### Surface trypsin digestion of *A. phagocytophilum* from infected HL60 human cells

The *A. phagocytophilum* human NY18 isolate was propagated in cultured HL60 human promyelocytic cells as previously described (de la Fuente et al., 2005). The *A phagocytophilum*-infected cells (~1 × 10^7^ cells) were collected when 70–80% of the cells were infected as determined by detection of intracellular morulae in stained cytospin cell smears. Host cell-free bacteria were isolated from cell lysates after five passages through a 27-gauge syringe, followed by differential centrifugation in Percoll gradients as previously described for *A. marginale* to separate bacteria from host cell debris (Lis et al., 2014). The pellet of purified *A. phagocytophilum* was resuspended in 200 μl of SPG buffer (0.25 mM sucrose, 10 mM sodium phosphate, 5 mM L-glutamic acid, pH 7.2), and 5 μl of sequencing-grade trypsin (Promega, Madison, WI, USA) was added to half of the cell reaction mixture. Bacteria were incubated at 37°C for 30 min and then centrifuged at 10,000 × g for 15 min and resuspended in Laemmli protein loading buffer, boiled for 5 min and loaded onto a 12% SDS-PAGE and analyzed by Western blot using rabbit antibodies against recombinant MSP4 and HSP70 proteins as previously described (Villar et al., 2015b).

### Tertiary models and protein-protein docking

The active *A. phagocytophilum* HSP70 and MSP4 proteins were modeled using I-TASSER (Zhang, 2008), and the unbound (apo)-HSP70 protein was modeled using Robetta (Kim et al., 2004). All tertiary models were optimized with the Schrödinger's Maestro Protein Preparation Wizard (Li et al., 2007). All steric clashes were resolved via minimization with the default settings in the Schrodinger's Maestro package. For the tertiary models, the Protein Preparation Wizard clusters at the highest degree of hydrogen bonding in equilibrium were used. Monte Carlo orientations were performed (100,000) for each cluster. The optimized structure is based on electrostatic and geometric scoring functions. The membrane positioning for MSP4 was calculated by the OPM database (Lomize et al., 2006) and generated using the Desmond systems builder (Bowers et al., 2006) as part of the Schrodinger's Maestro package. The protein-protein docking was assessed using the SwarmDock server (Torchala et al., 2013) that incorporates flexible docking by exploring in proximity to the Cartesian center of mass of the target protein. Minimization steps are included for the whole system. The poses are calculated based on the most energy favorable poses, minimized once again and sent to the user. We chose to analyze the top 10 poses since these were highly energy favorable (−41 to −54 kcal/mol). The server also produces the residue contacts made between both proteins. All structures were visualized and analyzed using the Visual Molecular Dynamics (Humphrey et al., 1996).

### Adhesion of recombinant *E. coli* strains to HL60 human cells

The adhesion of recombinant *E. coli* strains to HL60 human cells was characterized as previously reported (de la Fuente et al., 2001; Villar et al., 2015b). Briefly, *E. coli* strains producing *A. phagocytophilum* MSP4, HSP70, and mutant HSP70 with truncated peptide-binding domains that are involved in protein-protein interactions (Villar et al., 2015b) recombinant proteins were grown and induced as described before. The *E. coli* cells transformed with expression vector alone were used as negative control. Cell densities were determined and adjusted to 10^8^ cells per ml in Luria Broth (LB). One hundred microliters (10^7^ bacteria) culture were added to 900 μl of 10^6^ cells per ml suspensions of HL60 human cells in LB. Human cells and bacteria were incubated for 30 min at 37°C with occasional agitation. Cells were then collected by centrifugation, washed two times in PBS and resuspended in 100 ml of PBS. Elimination of unbound bacteria from human cells with bound bacteria was performed by Percoll (Sigma, St. Louis, MI, USA) gradient separation (de la Fuente et al., 2001). The band containing human cells was removed with a pipette and washed in PBS. The final cell pellet was lysed in 1 ml of sterile water and 5 μl plated onto LB agar plates containing 100 μg of ampicillin per ml. Two replicates were done for each experiment. Adhesive bacteria were quantitated as the number of colony forming units (CFU) recovered from each test and compared to the control values by Chi2 test (*P* = 0.001; *N* = 2).

### Transmission electron microscopy (TEM)

The HL60 human cells incubated as described above with *E. coli* strains producing *A. phagocytophilum* MSP4 and HSP70 recombinant proteins or transformed with expression vector alone as controls were pelleted and fixed in 2.5% glutaraldehyde in 0.1 M phosphate buffer for 1 h at room temperature. Fixed cells were washed three times in 0.1 M phosphate buffer with 4% glucose and embedded in 2% agar at 60°C. Samples were post-fixed using 2% OsO_4_ for 2 h at room temperature, three times washed and dehydrated in a graded series of acetone (30–100%) solutions for 15 min at each step. Samples were infiltrated with 25, 50, and 75% solutions of Spi Pon Epoxy resin (Structure Probe, Inc. Supplies, West Chester, PA, USA) diluted in anhydrous acetone for 1 h at each step. Samples were left in 100% resin overnight, transferred to embedding molds and polymerized at 60°C for 48 h. Ultrathin sections were contrasted in ethanolic uranyl acetate and lead citrate, carbon coated and observed in a JEOL 1010 TEM (JEOL Ltd., Akishima, Tokio, Japan) at an accelerating voltage of 80 kV. Images were captured using a Mega View III camera (Olympus Soft Imaging Solutions GmbH, Münster, Germany).

### Prediction of B-cell epitopes

*Anaplasma phagocytophilum* MSP4 (AFD54597) and HSP70 (AAC31306) amino acid sequences were aligned using MAFFT version 7, applying a gap opening penalty of 3 (range 1–3, default 1.53; Katoh and Standley, 2013). Sequence homology was calculated using Clustal Omega (Sievers et al., 2011). The linear B-cell epitopes on *A. phagocytophilum* MSP4 and HSP70 proteins were predicted using the Bepipred Linear Epitope Prediction tool (http://tools.immuneepitope.org/bcell/; Haste Andersen et al., 2006; Larsen et al., 2006; Ponomarenko and Bourne, 2007). Subsequently, to search for epitope homology, each predicted epitope within each protein was aligned with the full sequence of the other protein.

### Antibody inhibition assay

The inhibitory effect of rabbit IgG antibodies against MSP4 and HSP70 recombinant proteins on *A. phagocytophilum* human NY18 (Asanovich et al., 1997) and sheep (Alberdi et al., 2015) isolates infection of HL60 human cells was conducted as described previously for tick cells (Villar et al., 2015b). The inhibitory effect of IgG antibodies purified from MSP4 and MSP4-HSP70 immunized and control sheep at 0 and 94 days post-infection (dpi) on *A. phagocytophilum* human NY18 isolate infection of HL60 human cells was conducted using the same experimental approach as for rabbit IgG. The IgGs were purified from sheep sera using the NAb Protein G spin kit (Thermo Fisher Scientific, Waltham, MA, USA) following manufacturer's recommendations. HL60 cells were pooled and used to seed 24-well plates for each assay. Each well received 1 × 10^6^ cells in RPMI 1,640 medium (Gibco, Thermo Fisher, Madrid, Spain) 48 h prior to inoculation with *A. phagocytophilum*. Infected cultures for inoculum were harvested when infection reached 80% and host cells were mechanically disrupted with a syringe and 26-gauge needle. Rabbit or sheep purified IgGs (2.2–2.4 mg/ml) were mixed with inoculum (1:1) for 60 min before being placed on the cell monolayers. Each monolayer then received 100 μl of the inoculum plus IgG mix and plates were incubated at 37°C for 30 min. The inoculum was removed from the wells and cell monolayers washed three times with PBS. Complete medium (1 ml) was added to each well and the plates were incubated at 37°C. The control for each trial included inoculum incubated with rabbit pre-immune IgG or control sheep IgG. Four replicates were done for each treatment. After 7 days, cells from all wells were harvested, resuspended in 1 ml PBS and frozen at −70°C. Samples were thawed and solubilized with 1% Triton-X100 and processed for *A. phagocytophilum* detection by PCR after DNA extraction using TriReagent (Sigma) according to the manufacturer's recommendations. *Anaplasma phagocytophilum* infection levels were determined by *msp4* real-time PCR normalizing against human β*-actin* as described previously (de la Fuente et al., 2005) but using oligonucleotide primers MSP4-L (5′-CCTTGGCTGCAGCACCACCTG-3′) and MSP4-R (5′-TGCTGTGGGTCGTGACGCG3′), with PCR conditions of 5 min at 95°C and 35 cycles of 10 s at 95°C, 30 s at 55°C and 30 s at 60°C. Results were compared between treatments by the Student's *t*-test with unequal variance (*P* = 0.05; *N* = 4).

### Protein inhibition assay

HL60 cells were incubated with 4 μM MSP4 and HSP70 recombinant proteins or their combination in culture media for 1 h at 37°C and 5% CO2 in a humidified atmosphere. For antigen combination, equal molar ratios of each protein, equivalent to one part of HSP70 and two parts of MSP4, were incubated at 4°C on a rotator overnight. HL-60 cells (4 × 10^5^ cells/well) were fixed in 4% paraphormaldehyde (PFA) in PBS for 1 h at room temperature (RT), and then incubated with a 6x-His epitope tag monoclonal antibody (3 μg/ml mouse IgG1, Thermo Fisher 4A12E4) for 1 h at RT. After washing, cells were incubated with FITC-conjugated goat anti-mouse IgG (1/100, Sigma F2012) for 1 h at RT. Protein binding was assessed by flow cytometry using a FACScalibur® Flow Cytometer, equipped with the CellQuest Pro® software (BD-Biosciences, San Jose, CA, USA) as previously described (Seidman et al., 2015; Hebert et al., 2017). Incubation with PBS was used as negative control. The viable cell population was gated according to forward scatter and side scatter parameters. To determine the effect of MSP4 and HSP70 recombinant proteins on *A. phagocytophilum* infection, HL60 cells were incubated with 4 μM MSP4 and HSP70 recombinant proteins or their combination for 1 h, after which *A. phagocytophilum* human NY18 isolate bacteria purified as described above were added and incubated with the host cells in the continued presence of recombinant protein for 2 h. Unbound bacteria and proteins were removed and the infection was allowed to proceed for 48 h. Then, cells were harvested and infection levels determined by PCR and statistically analyzed as described above. The *Rhipicephalus microplus* Subolesin recombinant protein (SUB; Merino et al., 2011) and PBS were included as controls. Four replicates were done for each treatment.

### Lamb immunization and infection with *A. phagocytophilum*

The recombinant MSP4 was formulated alone or combined with HSP70. Antigen combination was done as described above in Section Protein Inhibition Assay. Recombinant antigens or saline control were adjuvated in Montanide ISA 50 V2 (Seppic, Paris, France; Merino et al., 2013). Nine 3-month old lambs of the Latxa breed (Basque Country, Spain) were selected from the experimental sheep flock maintained at NEIKER and were kept indoor during the experiment. This flock has no known history of ticks or tick-borne diseases. However, blood of lambs and their dams were analyzed prior to the start of the study to check their status for hemoparasites *Theileria, Babesia*, and *Anaplasma* spp. as previously described (Hurtado et al., 2015). All animals were negative for these hemoparasites. Three groups of 3 lambs each with similar live weight were formed. Lambs from each group were injected subcutaneously in the loose skin of the axilla (armpit) using a sterile syringe with removable needle 20 G × 1″ (9.0 × 25 mm), and taking aseptic precautions. Lambs were immunized three times on days 55, 30, and 10 before experimental infection with 1 ml doses of MSP4 (100 μg/dose), MSP4-HSP70 (100 μg of equal molar ratios of each protein/dose) or adjuvant/saline as control. The strain of *A. phagocytophilum* used for experimental infection originated from an infected lamb in Norway, which suffered TBF but was negative to other tick-borne pathogens (Alberdi et al., 2015; Stuen et al., 2015). The inoculum consisted of *A. phagocytophilum* infected heparinised blood that had been stored at −70°C with 10% dimethyl sulfoxide (DMSO). Once unfrozen, 1 ml of infected blood containing 1.8 × 10^6^ infected neutrophils per ml was intravenously inoculated into each experimental lamb through the jugular vein using sterile winged infusion sets with needle 21 G × 3/4″ (0.8 × 19 mm).

### Sheep samples and analysis

Whole blood and serum samples were collected from the jugular vein of each lamb previous to each immunization, daily starting on infection day during 10 days, and at weekly intervals until the end of the experiment at 94 dpi (Supplementary Table 1). Rectal temperatures were taken daily until 10 dpi and then weekly until 94 dpi (Supplementary Table 1). Lambs were also weighed periodically (Supplementary Table 1). Hematological analyses including leukocyte and erythrocyte cell counts, leukocyte cell differentiation (percent neutrophils, lymphocytes, monocytes and eosinophils), hemoglobin levels, hematocrit, mean cell volume (MCV), and mean corpuscular hemoglobin (MCH) were performed with an electronic counter (Hemavet 950, Drew, USA) in blood samples collected in EDTA-containing tubes (Supplementary Table 1). Blood smears stained with Giemsa stain were examined to investigate the presence of *A. phagocytophilum* in blood cells (Supplementary Table 1). At least 100 neutrophils were counted and examined to calculate the number of infected neutrophils per milliliter blood of each lamb throughout the experiment. The differential percent of *A. phagocytophilum*-infected neutrophils was calculated as the difference between values at different dpi and values at 3 dpi when infected neutrophils were first detected (Supplementary Table 1).

### Analysis of the antibody response in lambs

An indirect ELISA test was performed to detect IgG antibodies against MSP4 and HSP70 proteins in immunized and control lambs using serum samples collected before each immunization and at 0, 7, 10, and 94 dpi. High absorption capacity polystyrene microtiter plates were coated with 100 μl (0.01 μg/μl solution of purified recombinant MSP4 or HSP70 protein) per well in carbonate-bicarbonate buffer (Sigma). After an overnight incubation at 4°C, coated plates were blocked with 100 μl/well of blocking solution (5% skim milk in PBS). Serum samples or PBS as negative control were diluted (1:100, v/v) in blocking solution and 100 μl/well were added into duplicate wells of the antigen-coated plates. After an overnight incubation at 4°C, the plates were washed three times with a washing solution (PBS containing 0.05% Tween 20). A donkey anti-sheep IgG-peroxidase conjugate (Sigma) was added (diluted 1:1000 in blocking solution) and incubated at room temperature for 1 h. After three washes with washing solution, 100 μl/well of substrate solution (Fast OPD; Sigma) was added. Finally, the reaction was stopped with 50 μl/well of 3N H_2_SO_4_ and the optical density (OD) was measured in a spectrophotometer at 450 nm. Antibody titers were expressed as OD_450nm_ (OD_lambsera_ OD_PBScontrol_). The antigen-specific antibody response in immunized lambs was corroborated by ELISA using pooled sera collected at 0 dpi, but incubating sera with *A. phagocytophilum* purified from infected HL60 human cells as described above in Section Antibody Inhibition Assay. Results from rectal temperature and hematological analyses, differential percent of *A. phagocytophilum*-infected neutrophils and antibody titers were compared between immunized and control groups by two-way ANOVA test (*P* = 0.05; *N* = 3).

### Analysis of *A. phagocytophilum* DNA levels in lambs

For the analysis of *A. phagocytophilum* infection in lambs during the trial, DNA was extracted from 200 μl of blood using the QIAamp DNA Mini Kit (Qiagen, Hilden, Germany), including negative extraction controls every 9 samples. DNA was stored at −20°C until subsequent analysis. The presence of *Anaplasma* spp. was firstly determined using a real-time PCR assay for the screening of piroplasms and *Anaplasma* spp. (RTi-PCR1) that targets the 16S rRNA gene of *Anaplasma* spp. and the 18S rRNA gene of piroplasms of the genera *Theileria* and *Babesia* (Hurtado et al., 2015). The assay also includes an internal amplification control (IAC) to monitor for possible inhibition of the PCR reaction. All samples positive to *Anaplasma* spp. in the RTi-PCR1 were analyzed with a multiplex PCR assay that specifically amplifies the major surface protein 2 (*msp2*) gene of *A. phagocytophilum*, and the *msp4* gene of *Anaplasma ovis* (RTi-PCR2). Sequences of primers and probes, as well as details on cycling conditions were as reported previously (Hurtado et al., 2015). Analyses were performed in 20 μl volume reactions using an ABI PRISM 7500 Fast Sequence Detection System (Applied Biosystems, Foster City, CA, USA). For the quantitative analysis of *A. phagocytophilum* infection levels, DNA was extracted from 200 μl blood samples using Nucleospin 96 Blood (Machery-Nagel, Düren Germany). A quatitative real-time PCR was conducted on DNA samples using a Quantitect SYBR Green RT-PCR Kit and a Rotor Gene Q thermocycler (Qiagen, Inc. Valencia, CA, USA) following manufacturer's recommendations. A dissociation curve was run at the end of the reaction to ensure that only one amplicon was formed and that the amplicon denatured consistently in the same temperature range for every sample (Ririe et al., 1997). The DNA levels were normalized against sheep *aldolase B* using primers Ovi-ALDOB-F: CCCATCTTGCTATCCAGGAA and Ovi-ALDOB-R: TACAGCAGCCAGGACCTTCT, and the genNorm method (ddCT method as implemented by Bio-Rad iQ5 Standard Edition, Version 2.0; Livak and Schmittgen, 2001). Normalized Ct values were compared between immunized and control groups by Student's *t*-test with unequal variance (*P* = 0.05; *N* = 3).

## Results

### The *A. phagocytophilum* MSP4 and HSP70 proteins are localized on the bacterial membrane and involved in pathogen infection of HL60 human cells

**Figure 1:**
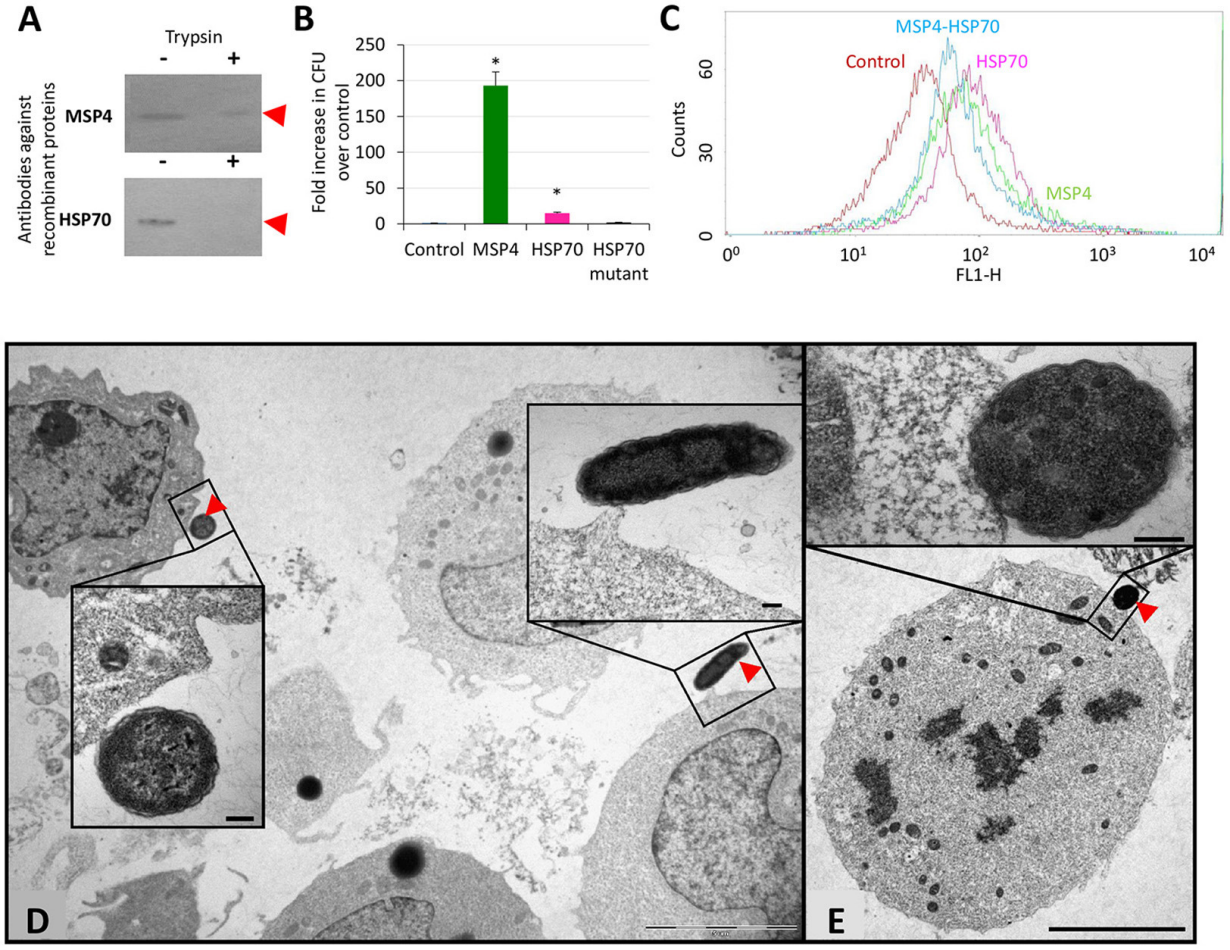
Role of *A. phagocytophilum* MSP4 and HSP70 proteins in interactions with HL60 human cells. **(A)** *A. phagocytophilum* (NY18) purified from infected HL60 human cells were mock treated (−) or surface digested with trypsin (+) and 10 μg protein loaded onto polyacrylamide gels for Western blot analysis using rabbit antibodies produced against recombinant proteins. These experiments have been repeated three times with similar results. **(B)** *E. coli* strains were grown and induced for the production of recombinant proteins. *E. coli* cells transformed with expression vector alone were used as negative control (Control). Bacteria adhesion to human HL60 cells was quantitated as the number of colony forming units (CFU) recovered from each test and compared to the control values by Chi2 test. Asterisks denote statistical significant differences between CFU recovered from *E. coli* transformed with MSP4 or HSP70 and the control (*P* < 0.001; *N* = 2 replicates per treatment). **(C)** HL-60 cells were incubated with *A. phagocytophilum* human NY18 isolate in the presence of recombinant MSP4, HSP70 and their combination. HL60 cells incubated with PBS served as negative controls. After washing to remove unbound bacteria and proteins, host cells were incubated for 48 h and binding of recombinant proteins to human host cells was analyzed by flow cytometry. The viable cell population was gated according to forward scatter and side scatter parameters. **(D,E)** Representative images of the adhesion of recombinant *E. coli* producing membrane exposed *A. phagocytophilum* proteins to human HL60 cells. **(D)** *E. coli* producing MSP4 or **(E)** HSP70 were incubated with HL60 cells and revealed by TEM to show adhesion to human cells (arrows). Cells incubated with control bacteria did not show adhesion to HL60 cells. Details of both interacting cells are shown in insets. Scale bars: 5 μm **(D,E)** and 200 nm (insets).

The subcellular localization of MSP4 and HSP70 proteins was characterized in *A. phagocytophilum* purified from infected HL60 human promyelocytic leukemia cells, mock treated or surface digested with trypsin and loaded onto polyacrylamide gels for Western blot analysis using rabbit antibodies specific against recombinant proteins. The results showed that as a transmembrane protein, MSP4 was partially resistant to trypsin digestion in *A. phagocytophilum* from HL60 cells, while HSP70 was extracellular and exposed to protease digestion (Figure 1A).

The apo and bound *A. phagocytophilum* HSP70 models showed that the major structural difference between the two HSP70 tertiary structures is at the C-terminus (residues 400–533), with a 40–90 Å α-carbon backbone root mean square deviation (RMSD; Supplementary Figure 1A). The *A. phagocytophilum* MSP4 showed a 25° ± 1° tilt from the membrane with its N/C-terminus oriented toward the cytosol, and the β-sheets buried within the membrane with remaining β-hairpin loops exposed extracellularly (Supplementary Figure 1B). The models suggested a limited number of possible MSP4-HSP70 binding positions due to the membrane orientation of MSP4 (Supplementary Figure 1C). The energy score of the optimum MSP4-HSP70 bound state (Supplementary Figure 1C) was calculated at −46 kcal/mol. Furthermore, although the residue map showed that the majority of protein-protein contacts are formed between the β-sheets of MSP4 buried within the membrane and the C-terminus of HSP70, several contacts between the β-hairpin loops of MSP4 and the N-terminus of HSP70 are exposed extracellularly, and therefore these residues may act as markers for mutational studies and antibody targeting (Supplementary Figure 1C). These models supported the interaction between *A. phagocytophilum* MSP4 and HSP70 proteins when localized on the bacterial membrane.

The binding of HSP70 and MSP4 to HL60 human cells was characterized using recombinant proteins and *E. coli* producing surface-exposed *A. phagocytophilum* proteins. The results demonstrated that MSP4 and HSP70 are involved in binding to human promyelocytic leukemia cells (Figures 1B,C). Furthermore, *E. coli* producing the mutant HSP70 with truncated peptide-binding domains that are involved in protein-protein interactions did not bind to human HL60 cells, thus supporting the role of this protein in interactions with host cells. The interaction of recombinant *E. coli* producing *A. phagocytophilum* MSP4 (Figure 1D) and HSP70 (Figure 1E) proteins with HL60 human cells was also characterized by electron microscopy in comparison with control *E. coli* cells to provide additional evidence for the role of these proteins in the interaction with vertebrate host cells.

**Figure 2:**
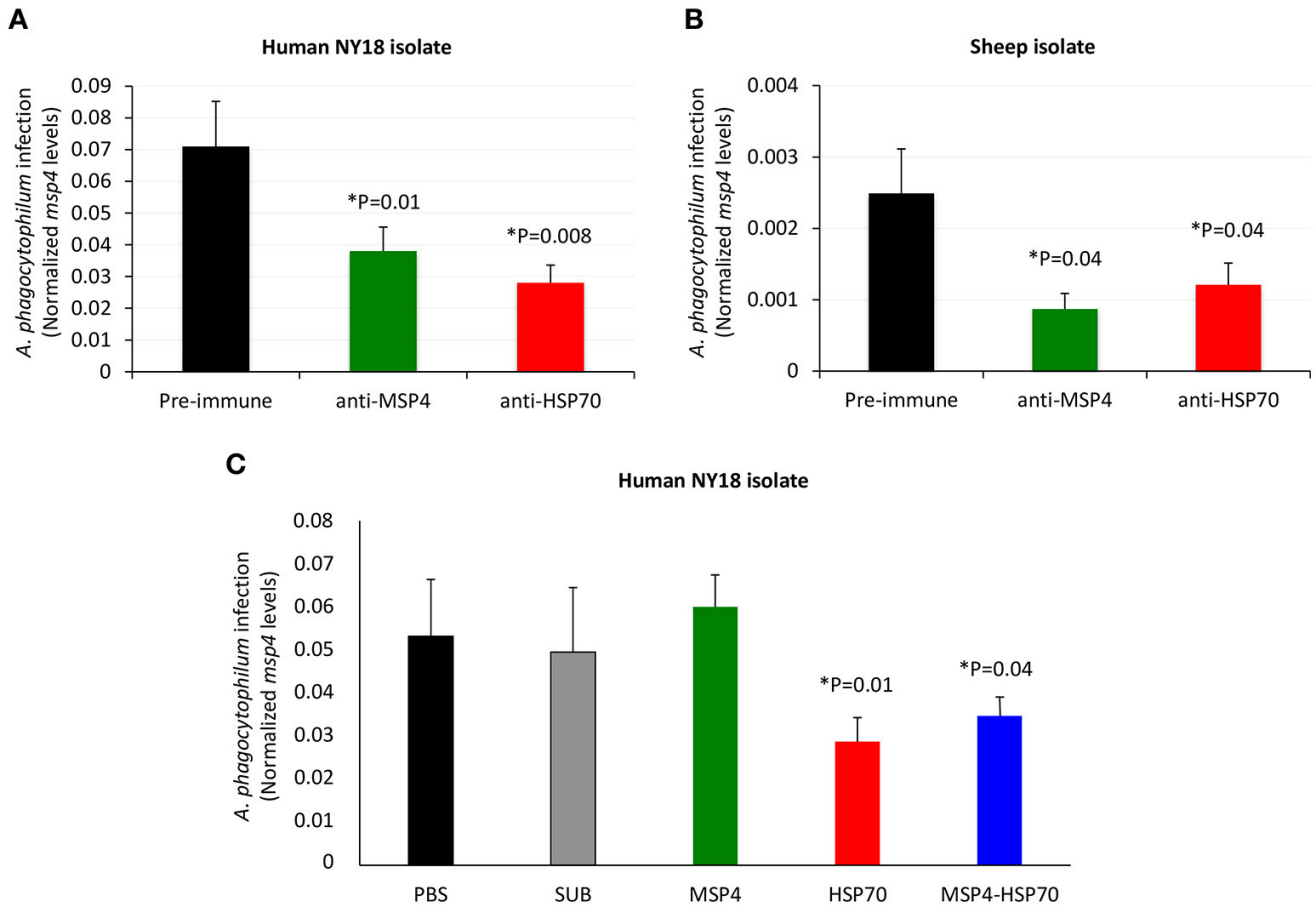
Role of *A. phagocytophilum* MSP4 and HSP70 proteins in infection of HL60 human cells. **(A,B)** Rabbit antibodies against *A. phagocytophilum* MSP4 and HSP70 recombinant proteins or **(C)** MSP4, HSP70 and MSP4-HSP70 recombinant proteins were used to characterize the inhibition of pathogen infection of HL60 human cells. Rabbit purified IgGs or recombinant proteins were mixed with *A. phagocytophilum* inoculum of human NY18 or sheep isolates for 60 min before being placed on the cell monolayers. Treatments included rabbit pre-immune serum, PBS and SUB as negative controls. *A. phagocytophilum* infection levels were determined by *msp4* real-time PCR normalizing against human β*-actin*. Results were compared between groups treated with pre-immune and anti-MSP4/HSP70 antibodies **(A,B)** or between groups treated with PBS or SUB and recombinant proteins **(C)** by the Student's *t*-test with unequal variance (^*^P < 0.05; *N* = 4 replicates per treatment).

To provide additional support for the role of *A. phagocytophilum* MSP4 and HSP70 proteins in the interaction with and infection of vertebrate host cells, recombinant proteins and antibodies against these proteins were used to evaluate their effect on pathogen infection of HL60 human cells. Anti-MSP4 and anti-HSP70 or recombinant MSP4 and HSP70 proteins were incubated with HL60 cells prior to infection with *A. phagocytophilum*. The results showed an inhibitory effect of anti-MSP4 and anti-HSP70 antibodies on infection of human cells with *A. phagocytophilum* human NY18 (Figure 2A) and sheep (Figure 2B) isolates when compared to cells treated with pre-immune serum. Furthermore, incubation with HSP70 and MSP4-HSP70 but not MSP4 recombinant proteins inhibited infection of human cells with *A. phagocytophilum* human NY18 isolate when compared to cells incubated with PBS or SUB controls (Figure 2C).

These results evidenced a role for MSP4 and HSP70 proteins in *A. phagocytophilum* adhesion to vertebrate host cells, and suggested a role for HSP70 during pathogen infection. These results also suggested that these proteins might constitute candidate protective antigens to prevent or control pathogen infection.

### Experimental infection with *A. phagocytophilum* correlates with TBF in lambs

To gain additional information on the role of *A. phagocytophilum* MSP4 and HSP70 proteins in host-pathogen interactions, sheep that are natural hosts for this pathogen were selected for immunization with recombinant proteins followed by experimental infection with *A. phagocytophilum*. Groups of three lambs each were immunized with recombinant MSP4, MSP4-HSP70 combination or adjuvant/saline control and infected with a sheep isolate of *A. phagocytophilum*. Then, several parameters including rectal temperature, animal weight, hemoglobin content, and hematological variables were evaluated in immunized and control *A. phagocytophilum*-infected lambs to correlate with TBF main clinical signs (Supplementary Table 1).

**Figure 3:**
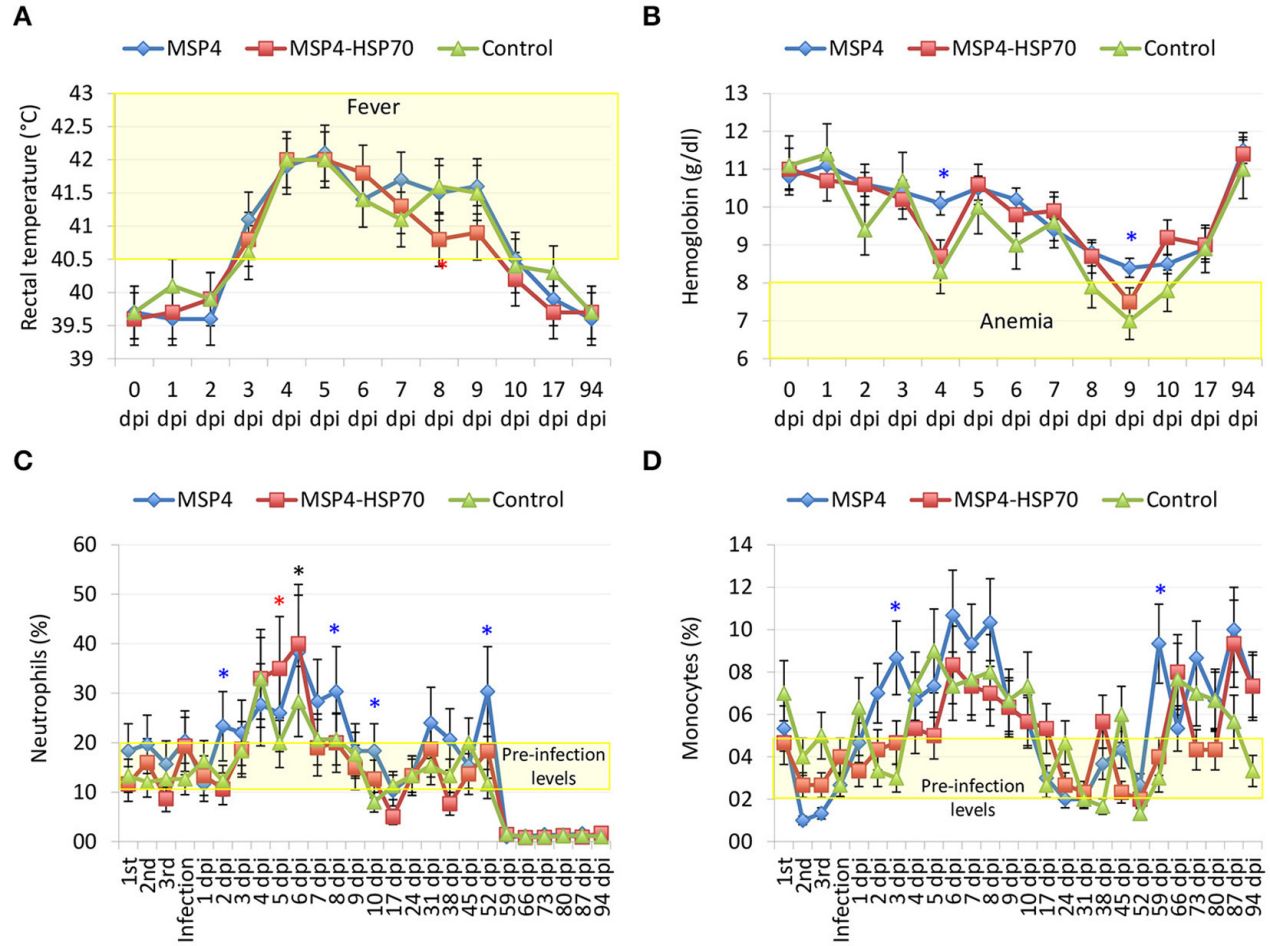
Evidence of TBF in lambs experimentally infected with *A. phagocytophilum* sheep isolate. Groups of three lambs each were immunized with recombinant MSP4, MSP4-HSP70 combination or adjuvant/saline control and experimentally infected with a sheep isolate of *A. phagocytophilum*. **(A)** Rectal temperatures were taken daily until 10 dpi and then weekly until 94 dpi. **(B–D)** Whole blood was collected in EDTA-containing tubes from the jugular vein of each lamb at different time points for different hematological analyses including **(B)** hemoglobin, and percent **(C)** neutrophils and **(D)** monocytes for leukocyte cell differentiation using an electronic counter (Hemavet 950, Drew, USA). Results from rectal temperature and hematological analyses were compared between immunized and control groups by two-way ANOVA test (^*^P < 0.05; *N* = 3 replicates per treatment). Red and blue asterisks denote statistical significant differences between MSP4-HSP70 and MSP4 immunized animals and controls, respectively.

The results showed signs of TBF in lambs infected with *A. phagocytophilum*. Evidence of *A. phagocytophilum* in neutrophils was obtained for all animals (Supplementary Table 1). Fever was evident in animals from all groups, primarily between 3 and 9 dpi (Figure 3A and Supplementary Table 1). Although immunized lambs tend to gain more weight, differences with controls were not significant (Supplementary Table 1). Control sheep showed evidence of anemia at 4 dpi, and between 8 and 10 dpi with all animals being anemic at 9 dpi, a result that correlated with low erythrocyte counts at 8–10 dpi (Figure 3B and Supplementary Table 1). The percent neutrophils in the leukocyte population increased in all animals after *A. phagocytophilum* infection between 4 and 9 dpi (Figure 3C and Supplementary Table 1). A severe neutropenia was evident in all animals after 59 dpi and lasted until the end of the experiment at 94 dpi (Figure 3C and Supplementary Table 1). Although monocyte levels were within normal values throughout the experiment, an increase was observed in all animals after infection between 2–10, 38–45, and 59–94 dpi (Figure 3D and Supplementary Table 1).

### The antibody response in immunized lambs is specific for *A. phagocytophilum* MSP4 and HSP70 recombinant proteins

**Figure 4:**
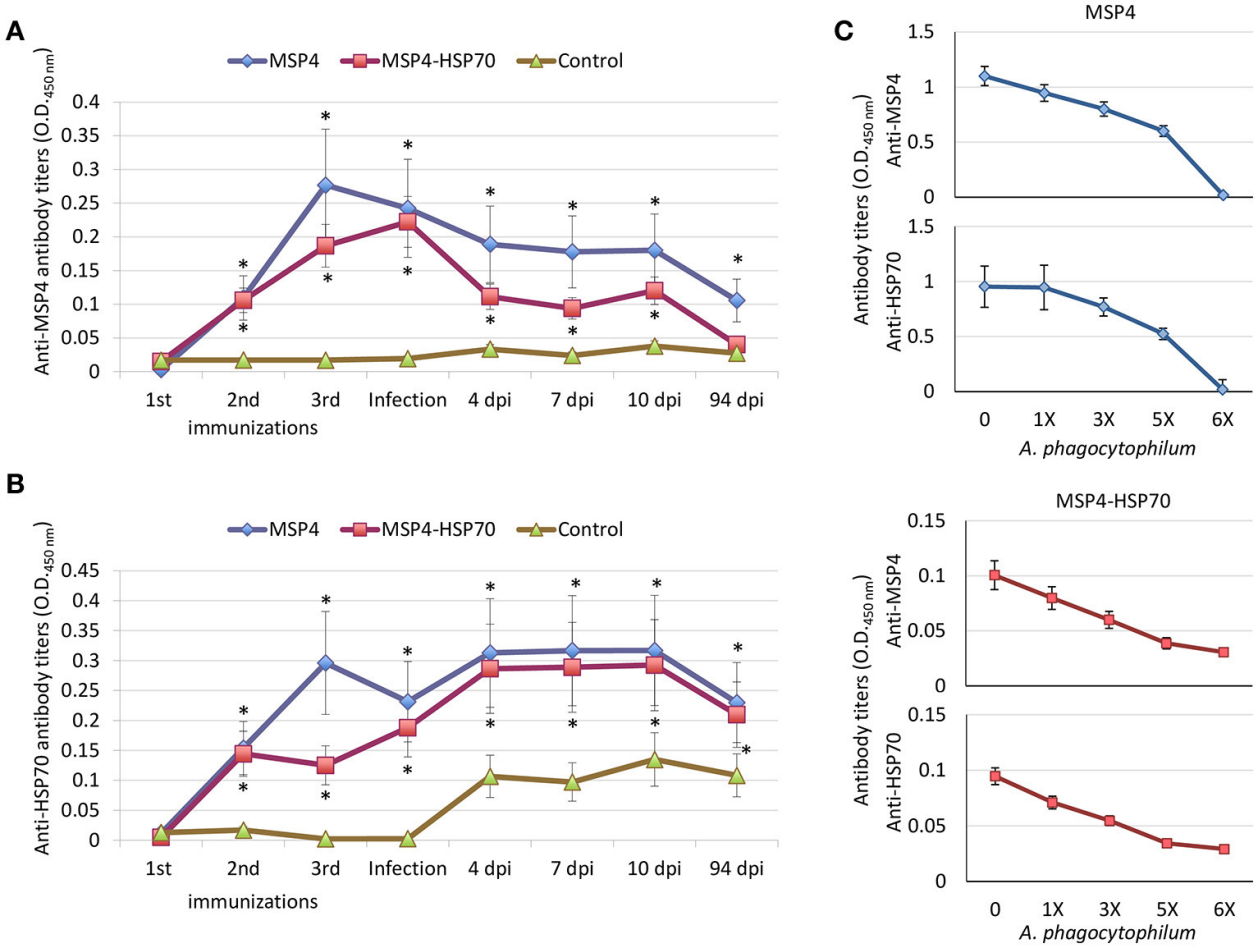
Antibody response in lambs immunized with *A. phagocytophilum* MSP4 and MSP4-HSP70 proteins. Groups of three lambs each were immunized with recombinant MSP4, MSP4-HSP70 combination or adjuvant/saline control and experimentally infected with a sheep isolate of *A. phagocytophilum*. An indirect ELISA test was performed to detect IgG antibodies against **(A)** MSP4 and **(B)** HSP70 proteins in immunized and control lambs using serum samples collected before each immunization and at 0, 7, 10, and 94 dpi. Antibody titers were expressed as OD_450nm_ (OD_lambsera_−OD_PBScontrol_). The results were compared between immunized and control groups by two-way ANOVA test (^*^P < 0.05; *N* = 3 replicates per treatment). **(C)** The antigen-specific IgG antibody response in immunized lambs was corroborated by ELISA using pooled sera collected at 0 dpi, but incubating sera with different concentrations of *A. phagocytophilum* purified from infected HL60 human cells.

The results showed that anti-MSP4 (Figure 4A) and anti-HSP70 (Figure 4B) IgG antibody titers were higher in MSP4-immunized than in control animals from second immunization until 94 dpi. In MSP4-HSP70 immunized lambs, anti-MSP4 IgG antibody titers were significantly higher until 10 dpi (Figure 4A), while anti-HSP70 IgG antibody titers remained higher then in control animals until 94 dpi (Figure 4B). The increase in the IgG antibody response to *A. phagocytophilum* MSP4 and HSP70 proteins after experimental infection was higher in immunized than in control animals (Figures 4A,B), suggesting an anamnestic response that may be protective against pathogen infection.

The IgG antibody response in MSP4 and MSP4-HSP70 immunized lambs was specific for MSP4 and HSP70 proteins as supported by the dilution effect observed after incubating sera collected before infection (0 dpi) with *A. phagocytophilum* purified from infected HL60 human cells (Figure 4C). However, the antibody response in MSP4-immunized lambs raised a question regarding the anti-HSP70 response in these animals. Possible explanations to this question are the production of polyreactive antibodies, and the existence of common B-cell epitopes between *A. phagocytophilum* MSP4 and HSP70 proteins. The MSP4 and HSP70 proteins were aligned and the linear B-cell epitopes were predicted and aligned to both protein sequences (Supplementary Figure 2A). A total of 14 and 32 linear B-cell epitopes were predicted for MSP4 and HSP70, respectively. Only epitopes longer than 8 amino acids were included in further analysis, resulting in 5 and 12 linear B-cell epitopes identified in MSP4 and HSP70, respectively. Only one epitope of MSP4 (DGATGYAI) aligned without gaps to a region of HSP70 (DGQTAVTI) with 50% identity (Supplementary Figure 2A). Three epitopes from HSP70 (FNDAQRQATKDAGTI, AGIKDNSKV and SNCSTDTLQQ) aligned without gaps to regions of MSP4 (FVAVGRDATLTPDNF, AGIPASNRV and AVCACSLLIS), with 26, 44, and 20% identity, respectively. These results suggested that antibodies against MSP4 epitopes (i.e., DGATGYAI) could cross-react with a region of HSP70 (DGQTAVTI), thus explaining the anti-HSP70 response in MSP4-immunized lambs. Furthermore, these epitopes were highly conserved because *A. phagocytophilum* MSP4 and HSP70 protein sequences show a high homology between different strains (Supplementary Figures 2B–D). In 56 of the MSP4 sequences available containing this region, the B-cell epitope was conserved (Supplementary Figure 2D). This region was conserved in all HSP70 sequences available in GenBank (Supplementary Figure 2D).

### Immunization of lambs with *A. phagocytophilum* MSP4 and MSP4-HSP70 recombinant proteins is only partially protective against TBF

To address the role of *A. phagocytophilum* MSP4 and HSP70 proteins as potential targets for the development of vaccines for the control of pathogen infection in vertebrate hosts, their potential protective capacity was characterized in immunized lambs.

The IgG antibody levels to MSP4 and HSP70 antigens remained higher after infection in immunized animals when compared to controls (Figures 4A,B). Although all animals showed signs of fever after infection with similar fever relapses, rectal temperature decreased faster in lambs immunized with MSP4-HSP70 (Figure 3A). The anemia typical of TBF was evident in control sheep at 8–10 dpi, while in immunized animals it did not occur (MSP4 group) or was observed at 9 dpi only (MSP4-HSP70 group; Figure 3B). Erythrocyte counts were not affected in immunized animals (Supplementary Table 1). The analysis of leukocytes, lymphocytes and eosinophils showed lower values at various dpi in immunized animals when compared to controls (Supplementary Table 1). In contrast, neutrophil and monocyte levels were higher in immunized animals when compared to controls at different dpi (Figures 3C,D; Supplementary Table 1). These results showed that while immunized animals presented evidence of TBF such as fever and neutropenia, the response to immunization resulted in less severe anemia in response to infection.

**Figure 5:**
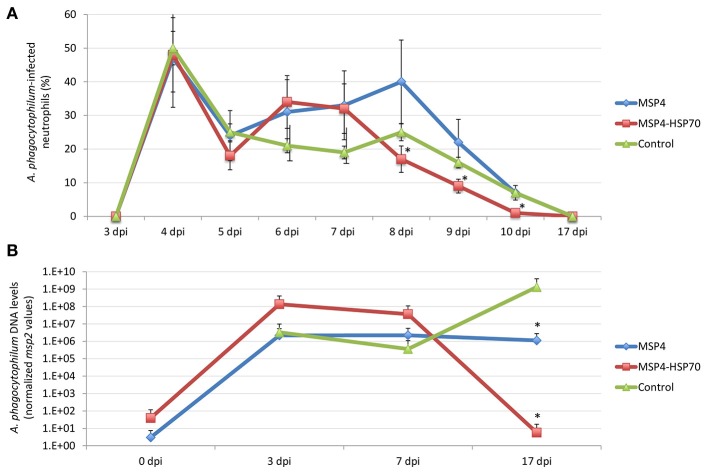
*Anaplasma phagocytophilum* infection levels in immunized and control lambs. **(A)** Blood smears stained with Giemsa stain were examined to investigate the presence of *A. phagocytophilum* in blood cells. At least 100 neutrophils were counted and examined to calculate the number of infected neutrophils per milliliter blood of each lamb. The differential percent of *A. phagocytophilum*-infected neutrophils was calculated as the difference between values at different dpi and values at 3 dpi when infected neutrophils were first detected. The results were compared between immunized and control groups by two-way ANOVA test (^*^P < 0.05; *N* = 3 replicates per treatment). **(B)** For the quantitative analysis of *A. phagocytophilum* infection levels, a quatitative real-time PCR was conducted. The *A. phagocytophilum* DNA levels were normalized against sheep *aldolase B* using the genNorm method (ddCT method as implemented by Bio-Rad iQ5 Standard Edition, Version 2.0). Normalized Ct values were compared between immunized and control groups by Student's *t*-test with unequal variance (^*^P < 0.05; *N* = 3 replicates per treatment).

Although the percent of infected neutrophils was apparently higher in immunized than in control animals at some dpi, the results suggested differences in the initial infection rate despite the injection of the same amount of unfrozen infected blood (Supplementary Table 1). These differences could be explained by variations in cell viability between different inoculums, resulting in animal-to-animal variations in the initial infection rate. Therefore, the differential percent of infected neutrophils with respect to the initial value at 3 dpi was used to characterize the effect of vaccination to normalize for these differences. The results showed a significant decrease in *A. phagocytophilum*-infected neutrophils in animals immunized with the MSP4-HSP70, but not MSP4 antigen at 8–10 dpi when compared to controls (Figure 5A). Furthermore, the *A. phagocytophilum* normalized DNA levels were significantly lower in lambs immunized with MSP4 and MSP4-HSP70 antigens at 17 dpi (Figure 5B). Taken together, these results suggested that the number of infected neutrophils decreased at 8–10 dpi in response to immunization with MSP4-HSP70, while pathogen levels per cell were lower in immunized lambs when compared to controls at 17 dpi.

### The antibodies against recombinant proteins in immunized lambs do not inhibit the *A. phagocytophilum* infection of HL60 human cells

**Figure 6:**
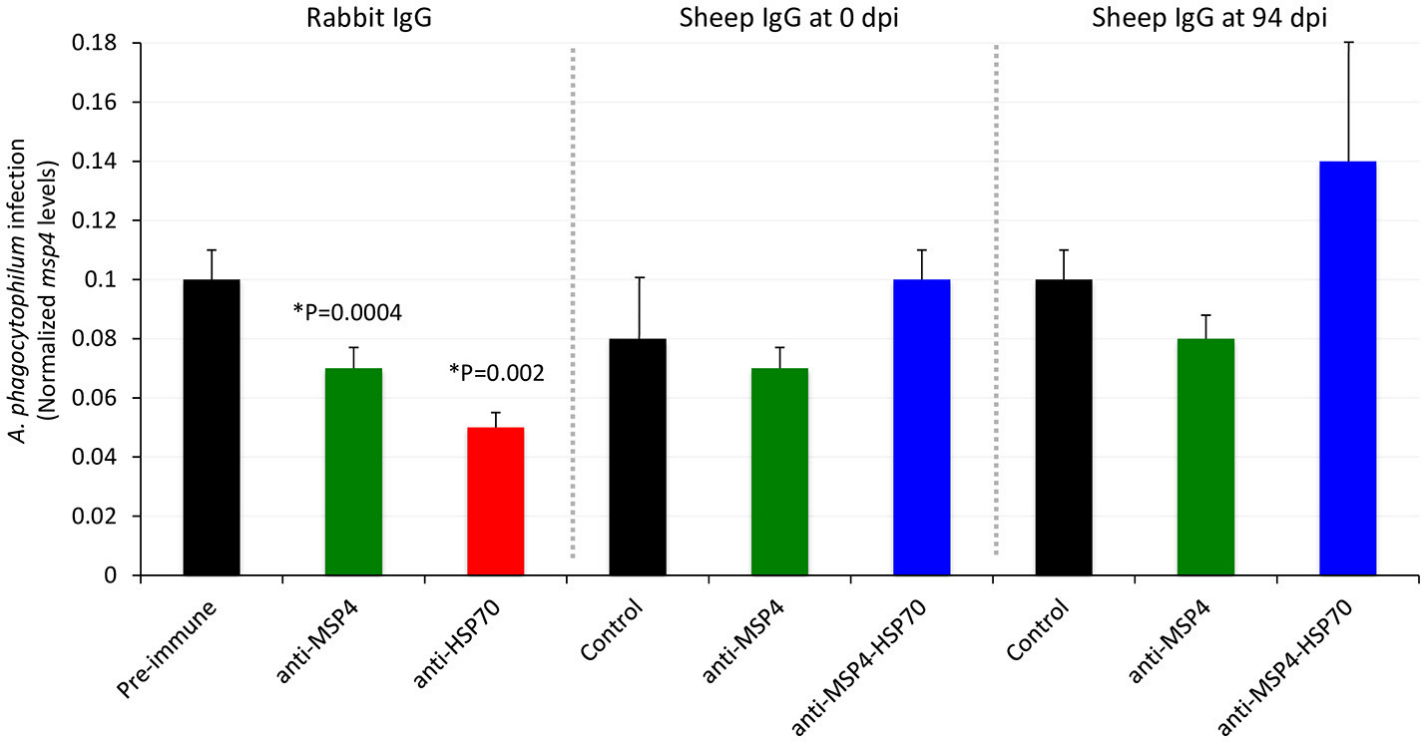
Role of antibodies against recombinant proteins from immunized lambs in the inhibition of *A. phagocytophilum* infection of HL60 human cells. Sheep IgG antibodies against *A. phagocytophilum* MSP4 and MSP4-HSP70 recombinant proteins were obtained from control and immunized sheep at 0 and 94 dpi and used to characterize the inhibition of pathogen infection of HL60 human cells. Treatments included purified IgGs from rabbit pre-immune, anti-MSP4 and anti-HSP70 sera. Purified IgGs were mixed with *A. phagocytophilum* inoculum of human NY18 isolate for 60 min before being placed on the cell monolayers. *A. phagocytophilum* infection levels were determined by *msp4* real-time PCR normalizing against human β*-actin*. Results were compared between groups treated with pre-immune/control and anti-MSP4/HSP70 antibodies by the Student's *t*-test with unequal variance (^*^P < 0.05; *N* = 4 replicates per treatment).

**Figure 7:**
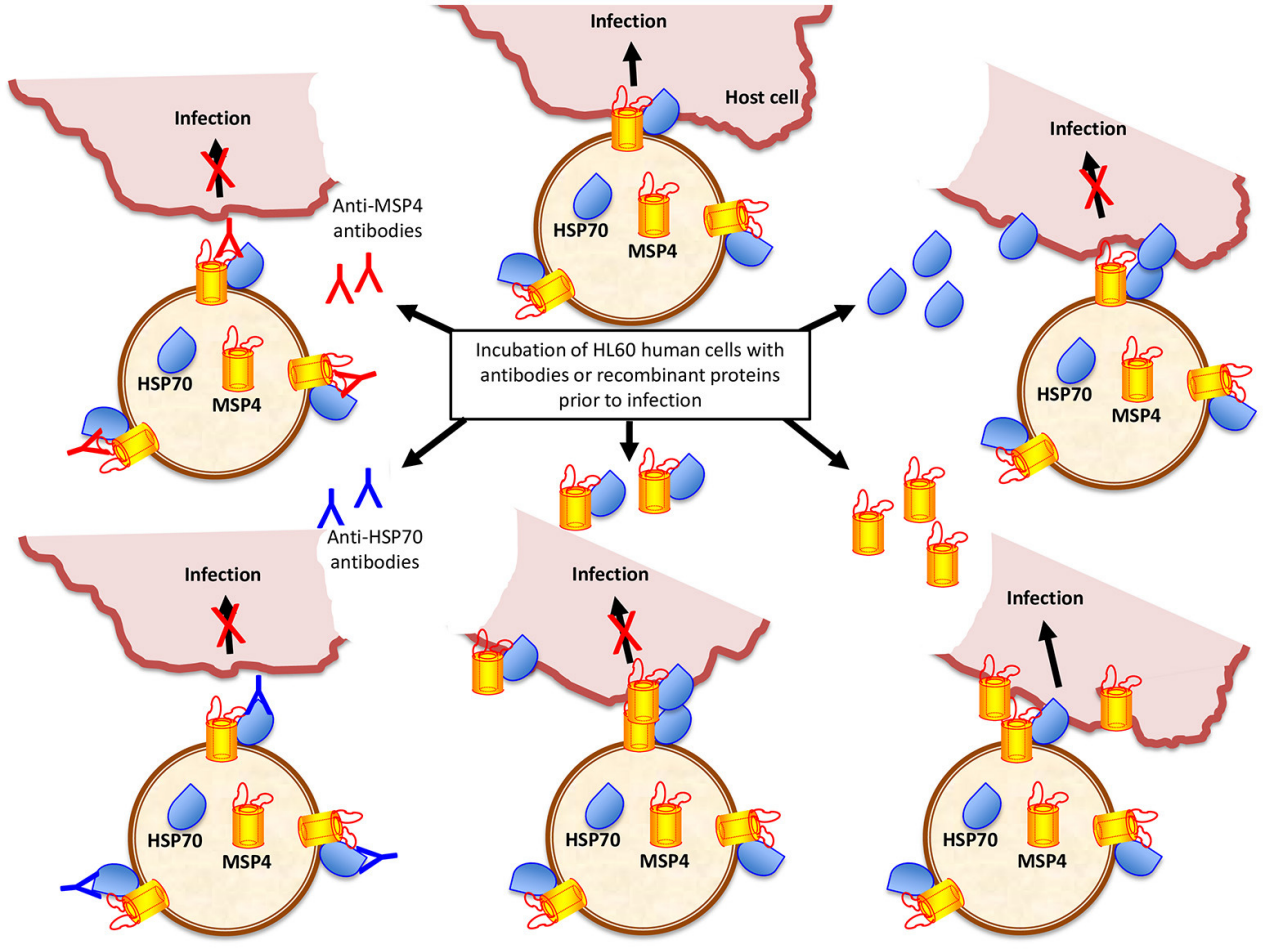
*Anaplasma phagocytophilum* HSP70 and MSP4 are necessary for pathogen infection of host cells. Based on the results of this study, a model was developed to explain the role of HSP70 and MSP4 during pathogen infection of host cells. HSP70 and MSP4 form a complex on the bacterial membrane where MSP4 probably acts a doking protein for HSP70. The incubation of HL60 human cells with recombinant HSP70 or MSP4-HSP70 interacting proteins inhibits infection by interfering with pathogen interaction with host cells mediated by HSP70, which is necessary for infection. However, the addition of recombinant MSP4 does not affect infection because the interaction of bacterial HSP70 with host cells occurs and is sufficient for infection. The anti-MSP4 antibodies probably inhibit infection through binding to MSP4 at the MSP4-HSP70 interaction site, thus preventing HSP70 adhesion to host cells. The anti-HSP70 antibodies bind to HSP70 and prevent the interaction with host cells required for pathogen infection.

An antibody inhibition assay using IgG from immunized sheep at 0 and 94 dpi was conducted to further characterize the antibody response in immunized lambs in relation with the protective capacity of MSP4 and MSP4-HSP70 antigens (Figure 6). While rabbit IgG antibodies against *A. phagocytophilum* MSP4 and HSP70 recombinant proteins inhibited pathogen infection of HL60 human cells (Figures 2A, 7), sheep IgG collected from immunized animals before infection (0 dpi) and after infection at the end of the experiment (94 dpi) did not affect pathogen infection (Figure 6). These results evidenced differences in the IgG response between immunized rabbits and lambs, and provided support for the limited protection against *A. phagocytophilum* infection observed in sheep immunized with MSP4 and MSP4-HSP70.

## Discussion

*Anaplasma phagocytophilum* transmembrane and surface proteins are involved in infection of vertebrate host cells (Seidman et al., 2015; Truchan et al., 2016). The *A. phagocytophilum* MSP4 and HSP70 proteins were previously shown to interact when localized on the bacterial membrane, with a possible role during pathogen infection of tick cells (Villar et al., 2015b). These results, together with the finding that *A. phagocytophilum* evolved common molecular mechanisms to establish infection in tick vectors and vertebrate hosts (de la Fuente et al., 2016a), suggested the hypothesis that MSP4 and HSP70 proteins have similar functions in host-pathogen and tick-pathogen interactions with possible implications as potential targets for the development of vaccines for the control of pathogen infection in both ticks and vertebrate hosts.

To address this hypothesis, we first characterized the role of these bacterial proteins in the infection of vertebrate host cells. The results using *A. phagocytophilum* derived from infected HL60 human cells corroborated those previously obtained with *A. phagocytophilum* derived from ISE6 tick cells (Villar et al., 2015b). The results showed that MSP4 is a transmembrane protein in *Anaplasma* spp. (de la Fuente et al., 2001), while HSP70 was probably translocated to the cell surface by still unknown mechanisms in which the bacterial type IV secretion system (T4SS) may be involved (Niu et al., 2006; Lin et al., 2007; Villar et al., 2015b). The binding of HSP70 and MSP4 to HL60 human cells was characterized using two alternative models based on recombinant proteins and *E. coli* producing surface-exposed *A. phagocytophilum* proteins with similar results, therefore supporting their role in the interaction with host cells. Although it is possible that the production of *A. phagocytophilum* proteins in *E. coli* may alter bacterial surface to cause binding to HL60 human cells not mediated by MSP4 and HSP70 proteins, previous results using this system with *A. marginale* MSP1a and MSP1b (de la Fuente et al., 2001) and with *A. phagocytophilum* proteins in tick cells (Villar et al., 2015b) makes this possibility unlikely. Furthermore, *E. coli* producing the mutant HSP70 with truncated peptide-binding domains that are involved in protein-protein interactions (Villar et al., 2015b) did not bind to human HL60 cells, therefore supporting the role of this protein in interactions with host cells.

Protein models supported the interaction between *A. phagocytophilum* MSP4 and HSP70 proteins when localized on the bacterial membrane, which was previously demonstrated in tick cells and may be functionally relevant for pathogen infection of both tick and vertebrate host cells (Villar et al., 2015b). Antibody inhibition assays showed that as previously discussed in the experiments using ISE6 tick cells (Villar et al., 2015b), antibodies against MSP4 and HSP70 proteins could affect the interaction between bacterial ligands and tick receptors to interfere with infection or affect the interaction with proteins functionally important for bacterial infection and/or multiplication in host cells. However, the inhibition assay using recombinant proteins suggested different roles for HSP70 and MSP4 during pathogen infection of host cells (Figure 7). While HSP70 seems to be directly involved in the pathogen interaction with host cells, MSP4 may acts as a doking protein for HSP70 to form the MSP4-HSP70 complex on the bacterial membrane (Figure 7). These results extended previous findings in tick cells (Villar et al., 2015b), supporting the role of MSP4 and HSP70 proteins in *A. phagocytophilum* infection and/or adhesion to vertebrate host cells.

The role of *Anaplasma* MSPs and other outer membrane proteins and invasins in adhesion to tick and vertebrate host cells for bacterial infection has been demonstrated in *A. marginale* and *A. phagocytophilum* (de la Fuente et al., 2001; Garcia-Garcia et al., 2004; Ge and Rikihisa, 2007; Rikihisa, 2011; Ojogun et al., 2012; Severo et al., 2012, 2013; Kahlon et al., 2013; Seidman et al., 2015; Truchan et al., 2016; Hebert et al., 2017). This mechanism appears to be conserved in other tick-borne pathogens, and in pathogen interactions with other arthropod vector species (de la Fuente et al., 2017). HSP70 was shown to relocate to the *Bacillus subtilis* membrane to restore membrane structure and function after ethanol stress (Seydlová et al., 2012), and to function in the molecular processing of *Borrelia burgdorferi* flagellin (Scopio et al., 1994). This protein may be functionally relevant at the *A. phagocytophilum*-host interface, and may interact with other membrane proteins for its function during pathogen infection (Susin et al., 2006; Multhoff, 2007).

To evaluate the potential protective capacity of these proteins, lambs that are natural hosts for this pathogen were immunized with recombinant MSP4, MSP4-HSP70 combination or adjuvant/saline control and infected with a sheep isolate of *A. phagocytophilum*. The MSP4-HSP70 combination was included based on evidence of protein-protein interactions, suggesting a physical and/or functional connection between these proteins (Villar et al., 2015b).

The results evidenced signs of TBF such as fever, anemia, and neutropenia in lambs infected with *A. phagocytophilum*, therefore validating the model for the comparative analysis between immunized and control animals. In sheep and dogs, *A. phagocytophilum* infection is accompanied by fever of approximate 7 days duration, which is the main clinical sign of TBF (Eberts et al., 2011; Stuen et al., 2011; Severo et al., 2013). The severe leukopenia and especially the prolonged neutropenia that accompanies the disease are also evident with TBF (Eberts et al., 2011; Stuen et al., 2011; Severo et al., 2013). Immune suppression by impaired antibody and lymphocyte response and reduced oxidative burst, together with anemia and monocytosis have been also reported in animals infected with *A. phagocytophilum* (Whist et al., 2003; Eberts et al., 2011). Weaning weight is also affected in lambs infected with *A. phagocytophilum* (Grøva et al., 2011).

The immunized lambs raised an antibody response that was specific for *A. phagocytophilum* MSP4 and HSP70 recombinant proteins. However, MSP4-immunized lambs developed an anti-HSP70 response. One possible explanation to this finding was the production of polyreactive antibodies, which constitute a major component of the natural antibodies that bind with low affinity to structurally unrelated antigens with broad antibacterial activity (Gunti and Notkins, 2015). Additionally, the analysis of protein sequences showed the existence of common B-cell epitopes between *A. phagocytophilum* human isolate MSP4 and HSP70 proteins that may also contribute to serum cross-reactivity. The B-cell epitopes are protein regions that bind to antibodies. Most epitopes are composed of different parts of the polypeptide chain that are brought into spatial proximity by the three-dimensional structure of the protein. These discontinuous epitopes can also react with continuous peptide fragments (i.e., linear epitopes) within the protein (Larsen et al., 2006). Epitopes can be understood as “antigenic determinants” within proteins and homology between linear epitopes can results in antibody cross-reactivity (Terajima et al., 2013).

Despite the effect of *A. phagocytophilum* infection on the impairment of antibody response in sheep (Whist et al., 2003), the results showed that IgG antibody levels to MSP4 and HSP70 antigens remained higher after infection in immunized animals when compared to controls. In contrast to the results reported in lambs immunized with inactivated *A. phagocytophilum* (Stuen et al., 2015), the number of fever relapses was similar between immunized and control animals, supporting that antigen-specific response is different from that obtained with whole organisms. The immunization with MSP4-HSP70 resulted in a decrease in the percent of infected neutrophils and pathogen levels per cell, supporting that immunization with MSP4-HSP70 was only partially protective for the control of *A. phagocytophilum* infection of neutrophils.

A previous experiment using a crude *A. phagocytophilum* protein extract for immunization did not protect against pathogen infection in sheep, but immunized lambs had reduced levels of infection (Stuen et al., 2015). The authors discussed that the lack of protection was probably due to the presence of not protective dominant antigens in the vaccine preparation, stressing the need for the identification of protective antigens conserved among different strains (Stuen et al., 2015). The results obtained here were similar to those reported previously by Stuen et al. (2015), but using two proteins shown to be highly conserved and involved in pathogen infection and/or interaction with host cells. The failure to protect lambs from *A. phagocytophilum* infection after immunization with MSP4 and MSP-HSP70 antigens may be due to several factors. Although these proteins seem to be involved in host-pathogen interactions and infection, other proteins may be also necessary for infection within this mechanism or as part of alternative mechanisms of infection. The results showed that IgG antibodies rose in immunized lambs did not inhibit *A. phagocytophilum* infection of HL60 human cells, suggesting differences between rabbit and sheep IgG responses that may be associated with epitope recognition in MSP4 and HSP70 proteins. These differences in the immune response between rabbits and sheep could be used to identify candidate protective regions or epitopes in MSP4 and HSP70 proteins to increase vaccine efficacy. Additionally, the intravenous inoculation of infected blood is different from natural infection after tick bite, and may affect the evaluation of the protective response after immunization.

## Conclusions

The *A. phagocytophilum* transmembrane and surface proteins play a crucial role during infection and multiplication in host neutrophils (Ge and Rikihisa, 2007; Rikihisa, 2011; Severo et al., 2012, 2013; Seidman et al., 2015; Truchan et al., 2016). However, the results reported here provided the first evidence for the role of *A. phagocytophilum* MSP4 and HSP70 proteins in this process. These results suggested that while membrane-localized MSP4 and HSP70 were involved in *A. phagocytophilum* interaction with host cells, HSP70 was directly implicated in pathogen infection. As for other intracellular pathogens, cellular immunity is essential for an effective protection against infection by *Anaplasma* spp. (Palmer et al., 1999; Hajdušek et al., 2013; de la Fuente et al., 2016a; Shaw et al., 2017). However, previous experiments have provided evidence that antibodies to bacterial proteins have a protective effect on infected hosts (Kaylor et al., 1991; Messick and Rikihisa, 1994; Sun et al., 1997; de la Fuente et al., 2003; Gomes-Solecki, 2014; Stuen et al., 2015). The results obtained here showed that the *A. phagocytophilum* MSP4-HSP70 antigen was only partially protective against pathogen infection in sheep. This limited protection may be associated with several factors, including the recognition of non-protective epitopes by IgG from immunized lambs. Nevertheless, these antigens may constitute candidate protective antigens for the development of vaccines against TBF in combination with other antigens. Focusing on the characterization of host protective immune mechanisms and protein-protein interactions at the host-pathogen interface may lead to the discovery and design of new protective antigens (de la Fuente et al., 2016c,d). Additionally, proteins involved in tick-pathogen and host-pathogen interactions such as *A. phagocytophilum* MSP4 and HSP70 may be used to develop double effect vaccines targeting infection in both vertebrate hosts and tick vectors (de la Fuente and Contreras, 2015).

## Author contributions

Jd and CG conceived the study. MC, PA, LM, IF, MVa, MVi, and NA performed the experiments. MC, AG, and SS performed the vaccine trial. AC, MC, JV, and Jd performed data analyses. Jd and MC wrote the paper, and other coauthors made additional suggestions and approved the manuscript.

### Conflict of interest statement

The authors declare that the research was conducted in the absence of any commercial or financial relationships that could be construed as a potential conflict of interest.
