# Supplementary material for: Anaplasma phagocytophilum MSP4 and HSP70 Proteins Are Involved in Interactions with Host Cells during Pathogen Infection
Source: Front Cell Infect Microbiol. 2017 Jul 5;7:307. doi: 10.3389/fcimb.2017.00307 (PMC5496961; doi:10.3389/fcimb.2017.00307)
Supplement: Supplementary file 2 [file DataSheet2.PDF]

Supplementary Figures

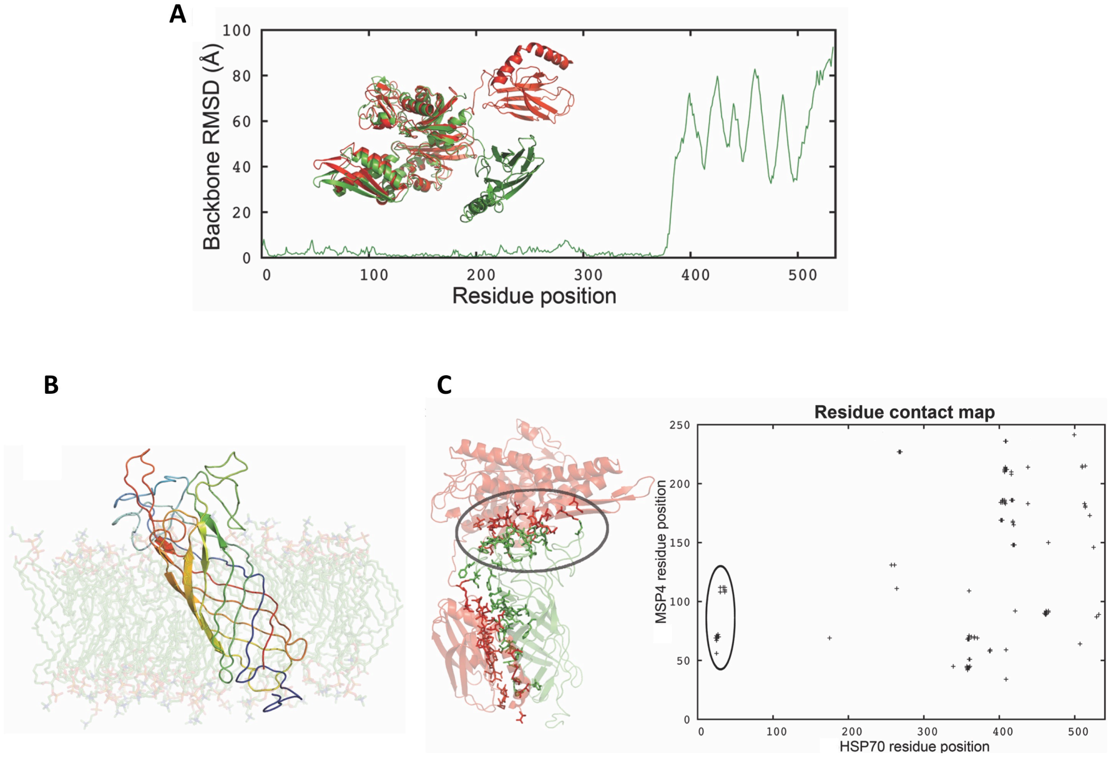

Supplementary Figure 1. Structural analysis and interactions of *A. phagocytophilum* MSP4 and HSP70 proteins. (A) Line graph on the α-carbon (backbone; y-axis) root mean square deviation (RMSD), for each residue (x-axis), between the tertiary structures of the apo-AHSP70 (red) and bound HSP70 (green). (B) Membrane-bound position and orientation of MSP4 that is color-coded from the N-terminus (blue) to the C-terminus (red). The membrane is shown as sticks and is atom color-coded (carbon = green; nitrogen = blue; oxygen = red; phosphorus = purple); hydrogen atoms are not shown. (C) HSP70 (red) bound to MSP4 (green) with their contact residues shown as predicted by SwarmDock. The Cartesian plot to the right is the all-reside contact map between the bound structures. The circles correspond to the tertiary (structure) and residue (plot) positions making contacts between the β-hairpin loops of MSP4 and the N-terminus of HSP70 that are exposed extracellularly, and therefore may act as markers for mutational studies and antibody targeting.

A

```
MSP4   M-------NYRELLVGSLSAAAVCACSLLISGSSFAYSGNNDASDVSG(13)PSFPSISS  62
HSP70  M(179)GDKQRTIVVYDLGGGTFDVSVLEIADGVFEVKATNGDTKLGG----EDFDNAIM  230
       *        : * ::* .*...:. .. * *:.. *  ...*. :.:.*     .* .

MSP4   FAISESDRGGSYVKGYNKNLSTLNVSDPASFTQHDPSFKFAKSLLTSFDGATGYAIGGAR  122
HSP70  EHMMESFQKETGINLRNDPMAVQRVKEAAEKAKIELSTRLETDITLPF--ISSDSTGAKH  288
         : ** :  : ::  *. ::. .*.:.*. :: : * :: ..:  .*   :. : *. :

MSP4   VEVEVGYKKFE----TLAESDYKHVESHNFVAVGRDATLTP------DNFF---------  163
HSP70  LSLKLSRAKFE(16)ALSDAGIKDNSKVDEVVLVGGMTRVPKVIQRVKDFFGKEPCQGVN  360
       :.:::.  ***     :*:::. *. .. : *.:  . * .*       .:**

MSP4   ---VMKIDSVKDISVMLNACYDVMHTDLPVSPYMCAGLGASFINIADHVTS---------  211
HSP70  PDEVVAVGAAIQGGILTGDVRDVLLLDVAPLSLGIETLGGVFTPLIERNTTIPTKKSQVF  420
           *: :.:. : .:: .    **:  *:.   .      **. *  : :: *:

MSP4   KLAYRGKVGVSY-------KLTPEISLIAGGSYHGI--- FDEQYAGIPASNRVNIAGGA  260
HSP70  STAEDGQTAVTIKVYQGERKMAIDNKLLGQFSLEGI(13)FDIDANGIVHVSAKDKASGK  489
       . *  *:..*:          *:: : .*:. * .**   ** :  **   .  : *.*

MSP4   AAKVK-----ANIASYGFNIG----ARFAFN  282
HSP70  EQTIKI(46)KSLKDYGDKVA(44)AAYAAN  603
          .:*      .: .** ::.     * :* *
```

B

Number of sequences	% Identity*
MSP4 (96)	
2	100
31	99-100
39	98-99
10	97-98
5	96-97
2	94-95
3	92-93
3	91-92
1	90-91
HSP70 (23)	
19	100
4	99-100

*Compared to the reference sequences AFD54597 (MSP4) and AAC31306 (HSP70).

C

```
                   Epitope
MSP4          110  DGATGYAI  118
HSP70         425  DGQTAVTI  432
                   ** *. :*
```

D

```
         ID             Epitope        Strains
MSP4   AFD54597   110  DGATGYAI  118     56
       ABS32272        EGATGYAI           3
       AAW32614        EGAAGYAI           1
       AEI91036        DGSTGYVI           1
                       :*::**.*

HSP70  AAC31306   425  DGQTAVTI  432     23
       KJV67926        DGQTAVTI
                       ********
```

Supplementary Figure 2. Comparison between MSP4 and HSP70 sequences. (A) Alignment between *A. phagocytophilum* MSP4 and HSP70 amino acid sequences. The number of amino acids in gap regions is shown in parenthesis. The B-cell epitope of MSP4 is underlined in blue. The region in HSP70 with the highest identify to the B-cell epitope of MSP4 is underlined in red. (B) Percent identity between MSP4 (N=96) and HSP70 (N=23) sequences available in GenBank. (C) Alignment between the B-cell epitope of MSP4 and the homolog region in HSP70. (D) Comparison of the B-cell epitope in MSP4 (N=61) and HSP70 (N=23) sequences available in GenBank that contain this region. For each variant, an ID of a reference sequence is provided.
